# Upadacinib for the Treatment of Refractory Morphea

**DOI:** 10.1111/ajd.14481

**Published:** 2025-04-03

**Authors:** Vincenzo Maione, Sara Rovaris, Carola Romanò, Stefano Bighetti, Iacopo Ghini, Luca Bettolini

**Affiliations:** ^1^ Department of Dermatology Spedali Civili, University of Brescia Brescia Italy; ^2^ Pathology Department University of Brescia, ASST Spedali Civili di Brescia Brescia Italy

**Keywords:** inflammatory skin disorders, JAK–STAT pathway, morphea, refractory skin conditions, upadacitinib

Dear Editor,

Morphea is an idiopathic inflammatory condition characterised by localised sclerotic hardening of the skin. First‐line treatments typically include topical corticosteroids and phototherapy, while more severe cases require immunosuppressive agents such as methotrexate and mycophenolate mofetil. However, therapeutic guidelines for refractory cases remain poorly defined.

A 77‐year‐old female patient presented to our clinic with infiltrated plaques on both legs, with more severe involvement of the left leg. Diagnosed with morphea in 2018 following a skin biopsy (Figure 1), her laboratory investigations, including antibody panels, were negative, and MRI ruled out linear or deeper morphea forms. The patient had no clinically relevant comorbidities. The patient underwent UVA1 phototherapy for three months, receiving two sessions per week, which yielded minimal improvement. Systemic corticosteroid therapy was initiated but discontinued due to serous chorioretinopathy, prompting a shift to a steroid‐sparing approach. The patient was administered methotrexate at a dose of 10 mg weekly for two months. Due to the onset of adverse effects, including nausea and fatigue, the dose was reduced to 5 mg weekly. However, these side effects persisted despite dose adjustment, and disease control remained inadequate, leading to treatment discontinuation. The patient's condition progressively worsened, especially in the left leg, with increased infiltration and hyperkeratotic skin changes.

**FIGURE 1:**
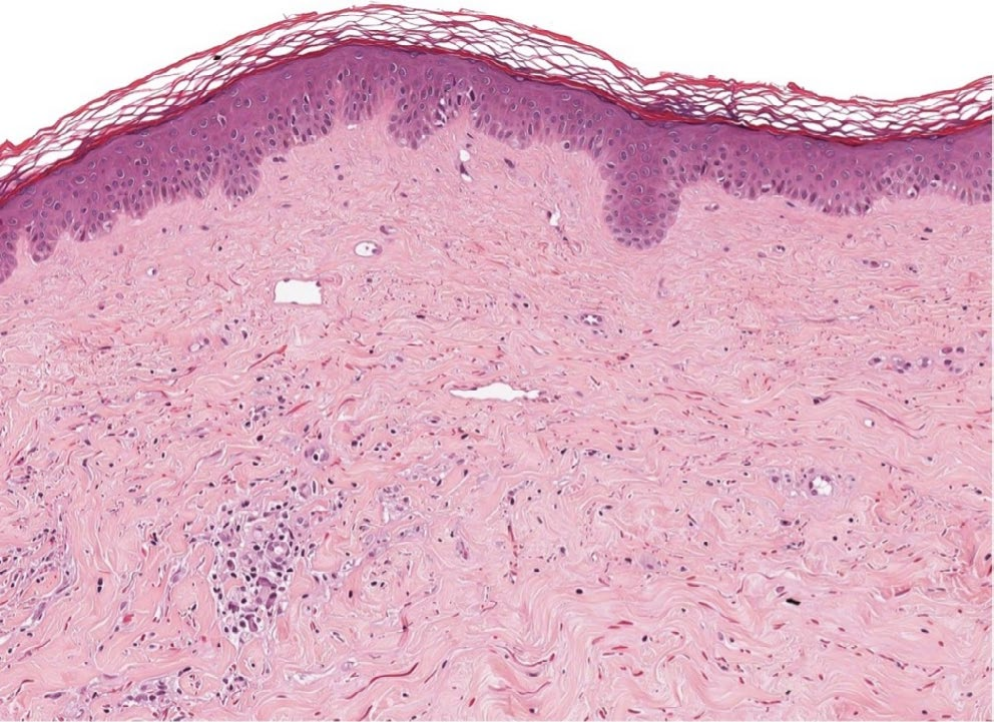
Histopathological examination revealing a full‐thickness fibrosclerotic dermis with a mixed inflammatory infiltrate, both superficial and deep, accompanied by the absence of pilosebaceous units and eccrine gland structures, with the epidermis maintaining a normotrophic and normokeratotic profile (EE, 40×).

Considering these developments, treatment was transitioned to mycophenolate. However, after three months, this therapy failed to effectively manage the active lesions in the left pretibial region, leading to significant induration and erosive‐ulcerative areas on erythematous‐edematous skin, prompting the discontinuation of this treatment (Figure 2a).

**FIGURE 2:**
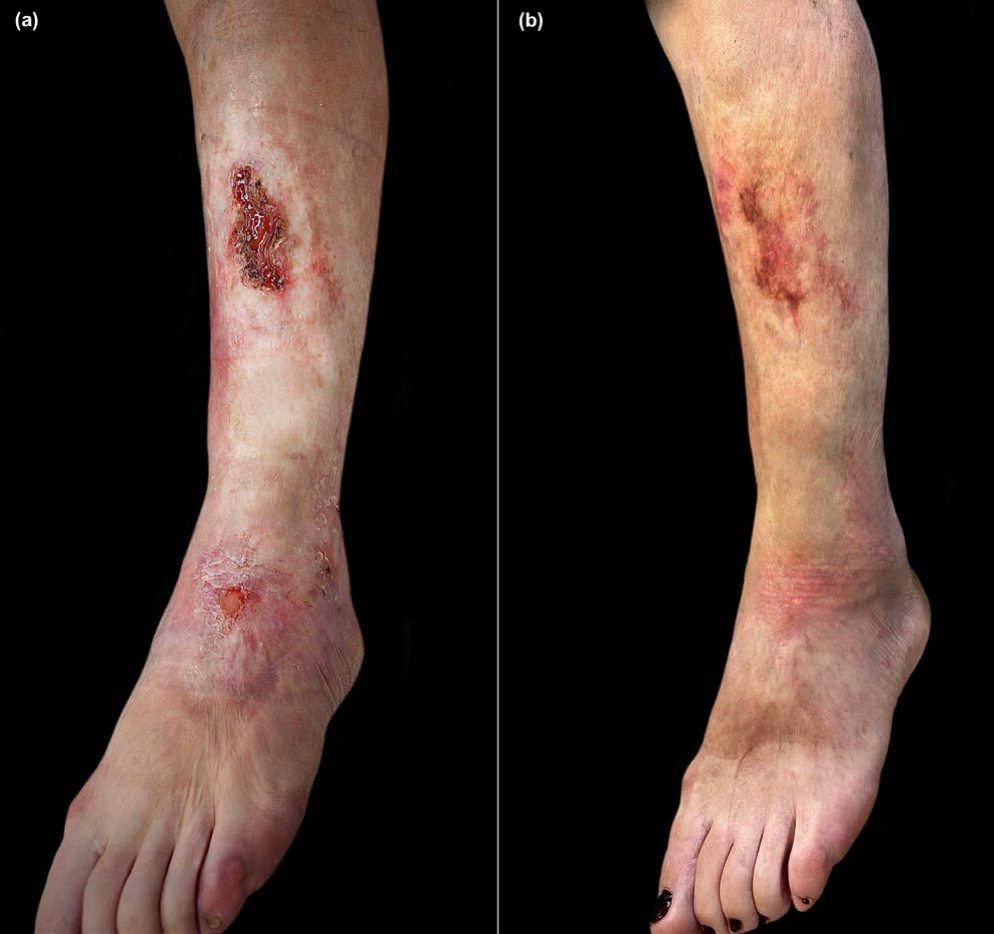
(a) Left leg of the patient demonstrating infiltrated plaques, significant induration, erosive‐ulcerative areas on erythematous‐edematous skin. (b) Isolated sclerotic patches, with resolution of ulcerative lesions and reduction of cutaneous induration at the 3‐month follow‐up.

Noninvasive techniques, including reflectance confocal microscopy and line‐field optical coherence tomography, were employed to monitor disease progression and exclude malignant transformation.

Subsequently, upadacitinib was administered at a dose of 15 mg per day. At the three‐month follow‐up visit, the patient exhibited significant improvement in her skin condition. Areas of normotrophic skin were observed in the pretibial region, with only isolated sclerotic patches remaining, and the ulcerative lesions had fully resolved (Figure 2b). Throughout this period, no significant modifications were noted in blood test results.

Upadacitinib is a JAK 1‐inhibitor that has received approval for use in adults to treat a variety of chronic inflammatory conditions, including moderate‐to‐severe rheumatoid arthritis, psoriatic arthritis, atopic dermatitis, ankylosing spondylitis, Crohn's disease and ulcerative colitis [1].

Given the JAK–STAT pathway's significant role in the inflammatory response associated with many immune‐mediated disorders, several studies have reported its efficacy in additional dermatological conditions, such as vitiligo, lupus erythematosus, hidradenitis suppurativa, dermatomyositis, lichen planus and sarcoidosis [1, 2, 3].

Morphea, a condition characterised by a complex pathogenesis, exhibits distinct inflammatory and sclerotic phases. During the inflammatory phase, there is an increased expression of the TH1/Th17 pattern, while the sclerotic phase is marked by a shift towards TH2 [4].

As a selective JAK 1 inhibitor, upadacitinib offers the potential to intervene across all pathogenic phases of morphea by inhibiting critical cytokines such as IFN‐gamma and IL‐6, which are integral to TH1 selection, as well as impairing the production of IL‐4 and IL‐6, which contribute to the sclerotic process. Notably, there have been documented cases in the literature of morphea resistant to conventional therapies, often treated with tofacitinib or baricitinib [5].

The distinct advantage of utilising upadacitinib lies in its favourable safety profile when compared to previous treatments, as evidenced by studies involving a range of cutaneous and noncutaneous diseases [1].

In conclusion, we present a case of refractory morphea that demonstrated an excellent response to upadacitinib, highlighting its potential role in managing more resistant forms of this condition. Further research is essential to validate our findings.

## Ethics Statement

The patients in this manuscript have given written informed consent to the publication of their case details.

## Conflicts of Interest

The authors declare no conflicts of interest.

## Data Availability

The data that support the findings of this study are available from the corresponding author upon reasonable request.

## References

[1] A. Agarwal , A. Diaz , R. Al‐Dehneem , R. M. Pineda , and S. Khattri , “Off‐Label Use of Janus Kinase Inhibitors in Inflammatory Cutaneous Diseases,” Journal of Drugs in Dermatology 22, no. 12 (2023): 1183–1190, 10.36849/JDD.7500. 38051858

[2] V. Maione , S. Bighetti , L. Bettolini , P. Incardona , and P. Calzavara‐Pinton , “Efficacy of Upadacitinib in a Case of Resistant Lupus Erythematosus Tumidus,” Journal of the European Academy of Dermatology and Venereology 38, no. 4 (2024): e335–e336, 10.1111/jdv.19604. 37909064

[3] V. Maione , S. Bighetti , S. Rovaris , S. Battocchio , P. Calzavara‐Pinton , and L. Bettolini , “A Case of Refractory Amyopathic Dermatomyositis Successfully Treated With Upadacitinib,” International Journal of Dermatology 63, no. 7 (2024): 959–961, 10.1111/ijd.17135. 38504645

[4] C. Papara , D. A. De Luca , K. Bieber , A. Vorobyev , and R. J. Ludwig , “Morphea: The 2023 Update,” Frontiers in Medicine (Lausanne) 10 (2023): 1108623, 10.3389/fmed.2023.1108623. PMC9969991 36860340

[5] W. Damsky , D. Patel , C. J. Garelli , et al., “Jak Inhibition Prevents Bleomycin‐Induced Fibrosis in Mice and Is Effective in Patients With Morphea,” Journal of Investigative Dermatology 140, no. 7 (2020): 1446–1449.e4, 10.1016/j.jid.2019.12.019. 31954727

